# Zeb1 Regulates E-cadherin and Epcam (Epithelial Cell Adhesion Molecule) Expression to Control Cell Behavior in Early Zebrafish Development*

**DOI:** 10.1074/jbc.M113.467787

**Published:** 2013-05-10

**Authors:** Corinne Vannier, Kerstin Mock, Thomas Brabletz, Wolfgang Driever

**Affiliations:** From the ‡ Developmental Biology, Institute Biology I, Faculty of Biology, Albert-Ludwigs-University Freiburg, Hauptstrasse 1, D-79104 Freiburg, Germany,; § Department of Visceral Surgery, University of Freiburg, D-79106 Freiburg, Germany, and; ¶ BIOSS-Centre for Biological Signalling Studies, Schänzlestrasse 18, D-79104 Freiburg, Germany

**Keywords:** Cell Adhesion, Development, E-cadherin, EMT, Transcription/Developmental Factors, Gastrulation

## Abstract

The ZEB1 transcription factor is best known as an inducer of epithelial-mesenchymal transitions (EMT) in cancer metastasis, acting through transcriptional repression of *CDH1* (encoding E-cadherin) and the EMT-suppressing microRNA-200s (miR-200s). Here we analyze roles of the ZEB1 zebrafish orthologs, Zeb1a and Zeb1b, and of miR-200s in control of cell adhesion and morphogenesis during gastrulation and segmentation stages. Loss and gain of function analyses revealed that Zeb1 represses *cdh1* expression to fine-tune adhesiveness of migrating deep blastodermal cells. Furthermore, Zeb1 acts as a repressor of *epcam* in the deep cells of the blastoderm and may contribute to control of epithelial integrity of enveloping layer cells, the outermost cells of the blastoderm. We found a similar ZEB1-dependent repression of *EPCAM* expression in human pancreatic and breast cancer cell lines, mediated through direct binding of ZEB1 to the *EPCAM* promoter. Thus, Zeb1 proteins employ several evolutionary conserved mechanisms to regulate cell-cell adhesion during development and cancer.

## Teaser

**Background:** Regulation of cell adhesion is important for embryonic development and to prevent cancer metastasis.

**Results:** Zeb1 controls cell adhesion in zebrafish embryos and human cancer cell lines through transcriptional repression of E-cadherin, Epcam, and miR-200s.

**Conclusion:** Zeb1 fine-tunes E-cadherin- and Epcam-mediated cell adhesion to control cell behavior during gastrulation.

**Significance:** Conserved cell adhesion regulation mechanisms are crucial for understanding development and cancer invasion.

## Introduction

Control of cell adhesion is essential for regulation of cell motility, organization of cells into tissues, and morphogenesis during development (1). Specifically, the transition of cells between epithelial and mesenchymal states is a hallmark of developmental morphogenetic events but may also initiate invasion and metastasis in cancer (2, 3). Complex regulatory networks controlling EMT^2^ have been described (4), but the precise contributions of specific molecular mechanisms to sophisticated morphogenetic events like gastrulation are not fully understood. Here, we investigated ZEB1 (δEF1, Zfhx1a, Tcf8), an EMT-inducing transcription factor of the zinc finger E-box binding homeobox family, with respect to its function during gastrulation and segmentation stages in early zebrafish development. ZEB1 is a key regulator of the EMT-factor network during tumorigenesis. Aberrant expression of ZEB1 in cancer cells induces EMT by repressing several cell-cell adhesion molecules, including E-cadherin (5, 6) and Plakophilin 3 (7), as well as basement membrane components (8) and cell polarity factors (9, 10). Beside the down-regulation of epithelial markers, EMT is characterized by an increased expression of mesenchymal markers, such as N-cadherin and vimentin (6). ZEB1-driven highly motile cancer cells show strong morphological plasticity; once they reach a secondary site they may be subject to a reverse morphological process, indicated by a mesenchymal-epithelial transition, that enables them to grow into overt metastases often resembling the epithelial tumor of origin (11, 12). Recently we and others have shown that this morphological plasticity of cancer cells is mediated by a double-negative feedback loop between ZEB1 and the miR-200 family members (13, 14).

In teleosts, the vertebrate *ZEB1* gene is represented by two paralogous genes, *zeb1a* and *zeb1b* (previously *kheper* (15)). Although it has been shown that Zeb1b is implicated in zebrafish gastrulation movements (15, 16), detailed functional and mechanistic analyses have not been performed. During zebrafish gastrulation four distinct cell movements act in concert to establish the three germ layers (17). During epiboly the initially static blastomeres become motile and spread over the yolk cell toward the vegetal pole. During emboly, presumptive mesendodermal cells migrate inwards at the vegetal margin underneath the epiblast to generate the hypoblast that will differentiate into mesendoderm. Convergence and extension movements condense cells of the embryo mediolaterally and elongate the body axis. Interestingly, in zebrafish all four gastrulation movements depend on tightly regulated E-cadherin-mediated cell-cell adhesion. Loss or gain of E-cadherin activity results in distinct changes of cell behavior before and during gastrulation (18–22).

Using Zeb1a and Zeb1b knockdown and overexpression in zebrafish embryos, we detected gastrulation defects, including severe epiboly retardation. Both Zeb1 paralogs have an important role during gastrulation in regulating adhesion of deep cells by repressing *cdh1* and *epcam* (epithelial cell adhesion molecule) expression. We also found a direct ZEB1-mediated repression of *EPCAM* in human pancreatic and breast cancer cell lines, indicating a conserved regulatory circuit. Finally, we show that Zeb1b represses transcription of miR-141 and -200b, two members of the miR-200 family. This finding and previously published data by Choi *et al.* (23) together reveal that the reciprocal ZEB1/miR-200 feedback loop, which plays an essential role in defining the EMT status and cellular plasticity of human cancer cell lines, is also conserved in teleosts. In the context of gastrulation, *zeb1* genes appear to contribute to fine-tuning of cell adhesion during the complex morphogenetic movements, in contrast to ZEB1 setting an EMT-like switch in cancer metastasis.

## EXPERIMENTAL PROCEDURES

### 

#### 

##### Zebrafish Strains and Maintenance

Fish maintenance was as described (24). Embryos injected with morpholinos (MOs) or specific mRNAs were staged according to morphology of their standard control MO (SCMO), control *gfp*, or control *nls-tomato* mRNA-injected littermates. All experiments were performed using zebrafish wild-type (WT) embryos of the AB/TL strain.

##### Whole-mount in Situ Hybridization (ISH) and Sectioning

ISH of whole-mount zebrafish embryos was performed as described (25) with minor modifications. The used digoxigenin-labeled (Roche Applied Science) riboprobes were generated using plasmids containing the DNA of interest (*cdh1* (26), RefSeq accession number NM_131820.1; *cdh2* (27), RefSeq accession number NM_131081.2; *egr2b* (28), RefSeq accession number NM_130997; *emx1* (29), RefSeq accession number NM_198144; *epcam* (30), RefSeq accession number NM_001017593.2; *gsc* (31), RefSeq accession number NM_131017.1; *ntla* (32), RefSeq accession number NM_131162.1; *pax2a* (33), RefSeq accession number NM_131184). For *zeb1a* a 435-bp fragment was amplified using the IMAGp998H2013118Q clone (Source BioSciences, Berlin) as template and gene-specific primers comprising T7 RNA polymerase promoters at their 5′ ends. The following primers were used: antisense probe, forward (5′-CCA TGT AAT ACG ACT CAC TAT AGG GCA GGT GCT CCT TCA GGT GAT GC-3′) and reverse (5′-GAG GAG TGC GTC AGT GAT GAG G-3′); sense probe, forward (5′-CAG GTG CTC CTT CAG GTG ATG C-3′) and reverse (5′-CCA TGT AAT ACG ACT CAC TAT AGG GGA GGA GTG CGT CAG TGA TGA GG-3′). PCR fragments were purified and directly used as templates for *in vitro* transcription with the T7 mMessage mMachine kit (Ambion). For *zeb1b* a 1621-bp fragment was amplified using the *pCS2*+*-zeb1b* plasmid (15) as template and gene-specific primers comprising T3 RNA polymerase promoters at their 5′ ends. The following primers were used: antisense probe, forward (5′-CAC AGC GAA AGG ATC ATG GCG GAT GG-3′) and reverse (5′-GCA TCA ATT AAC CCT CAC TAA AGG GAG ATC TTC AGA GGA GGC TGA CCA GGA CAC-3′); sense, forward (5′-GCA TCA ATT AAC CCT CAC TAA AGG GAG ACA CAG CGA AAG GAT CAT GGC GGA TGG-3′) and reverse (5′-TCT TCA GAG GAG GCT GAC CAG GAC AC-3′). PCR fragments were purified and directly used as templates for *in vitro* transcription with the T3 mMessage mMachine kit (Ambion). For sections, *in situ*-stained embryos were equilibrated in a gelatin/albumin mixture (0.49% gelatin, 30% BSA, 20% sucrose in PBS), transferred into freshly prepared polymerization solution (25% glutaraldehyde in gelatin/albumin solution, 7:100), and polymerized. 30-μm serial sections were taken using a Leica Vibratome VT1000S.

##### Embryo Injection

All antisense MOs were obtained from Gene Tools LLC. The injected antisense MOs included the *GeneTools* (Philomath) SCMO, *zeb1a/b* translational-blocking (TB) MO/*tcf8* MO (16), *zeb1a* TBMO (5′-GGG CCA TCC GCC ATG ATT TTT TGC A-3′), *zeb1b* splicing-blocking (SB) MO (5′-TTC TCC TGC ACA ACA CAA AAT GAA C-3′), located at the boundary of intron5/exon6, and *cdh1* TBMO/MO3-cdh1 (34). MOs against the miR-200 family are as published (23): anti-miR-141 (5′-GCA TCG TTA CCA GAC AGT GTT A-3′), anti-miR-200b (5′-GTC ATC ATT ACC AGG CAG TAT TA-3′), and anti-miR-429 (5′-ACGGCATTACCAGACAGTATTA-3′). MOs were injected into the yolk of one-cell stage embryos. Unless otherwise indicated, 4 ng of *zeb1a/b* TBMO, 8 ng of *zeb1a* TBMO, and 8 ng of *zeb1b* SBMO or the corresponding amount of SCMO per embryo were injected for knockdown studies. In the triple anti-miR-200 MO injection, 4 ng of each MO were co-injected into the yolk at the one-cell stage. Corresponding control embryos were injected with 12 ng of SCMO.

To check the specificity and efficacy of the *zeb1a/b* TBMO directed against the translation start site of *zeb1b*, *pCS2*+*-gfp-reporter* plasmids were created that harbor the *zeb1b* morpholino target sequence or the homologous region of the *zeb1a* gene, fused to the ATG-deleted ORF of the *gfp* gene. The *pCS2*+*-5′UTR-zeb1b-gfp* plasmid was linearized with Acc65I, the *pCS2*+*-5′UTR-zeb1a-gfp* plasmid was linearized with NotI. Both were transcribed using the SP6 mMessage mMachine kit (Ambion). The *gfp-reporter* mRNAs were injected into one-cell stage embryos with SCMO or the specific targeting morpholino along with *nls-tomato* mRNA. At epiboly stages, embryos were assayed for NLS-Tomato and GFP fluorescence (see also Fig. 2). To verify the efficacy of the *zeb1b* SBMO, one-cell stage embryos were injected with *zeb1b* SBMO or SCMO and allowed to develop until 75 % epiboly at which time RNA was isolated. RT-PCR was performed to detect misspliced mRNA (see also Fig. 4). To verify the efficacy of anti-miR-200 family MOs, one-cell stage embryos were injected with an anti-miR-200 MO mix (miR-141, -200b, -429) or SCMO, fixed at 48 h post fertilization (hpf), and assayed for miR-141, 200a/b/c, and -429 expression by whole-mount ISH.

The *zeb1b* expression construct was a kind gift of Masahiko Hibi (15). *In vitro* transcribed *gfp* or *nls-tomato* mRNA served as the injection control. The GFP expression construct in the pGI vector (kindly provided by Gudrun Aspöck) was linearized with NotI and transcribed using SP6 mMessage mMachine kit (Ambion). *nls-tomato* mRNA was generated using the (NLS)-Tomato in pDestTol2pA2 vector (Invitrogen) and the SP6 mMessage mMachine kit (Ambion). 50–100 pg of *zeb1b* mRNA or control mRNA (*gfp*, *nls-tomato*) per embryo were injected into the yolk at the one-cell stage or into a single blastomere at the four-cell stage.

##### shGFP and shZEB1 Clones

RNA isolation and cDNA synthesis of Panc-1 and MDA-MB231 shGFP and shZEB1 clones were described previously (14).

##### Cell Nuclei Isolation

Cell nuclei were isolated prior to RNA purification to reduce the amount of maternally deposited mRNAs. After enzymatic dechorionation, embryos (at least 120 per sample) were homogenized on ice by 1 stroke of a loose-fitting pestle (type A) and 4 strokes of a tight-fitting pestle (type B) in a Dounce glass homogenizer. The lysate was centrifuged at 107 × *g* for 5 min at 4 °C. The nuclei-containing supernatant was taken and centrifuged at 4816 × *g* for 5 min at 4 °C. The nuclei pellet was resuspended in 600 μl of RLT Plus buffer (Qiagen), briefly vortexed, and stored at −20 °C.

##### RNA Isolation

To isolate total RNA (including miRs) from whole embryos and nuclear extracts, we used the RNeasy® Plus Mini Kit from Qiagen, applying the Qiagen Supplementary Protocol: purification of miR from animal cells using the RNeasy® Plus Mini kit and RNeasy MinElute® Cleanup Kit (Protocol 1). To gain total RNA, up to 30 embryos in 600 μl of RLT Plus buffer were disrupted and homogenized by passing through a needle (diameter 0.60 mm) fitted to a RNase-free syringe. The homogenized probe was transferred to a QIAshredder column and centrifuged for 1 min at 10,000 rpm. Nuclear extracts in RLT Plus buffer were thawed and directly transferred to a genomic DNA eliminator spin column without any further disruption or homogenization. Total RNA containing miRs was finally eluted in 30–40 μl of RNase-free water.

##### Quantitative Real-time PCR (qRT-PCR)-mRNA

Reverse transcription of RNA was performed using the SuperScript^TM^ III Reverse Transcriptase kit from Invitrogen according to the manufacture's manual. For each zebrafish sample, 300 ng of total RNA were transcribed using 200 ng of random primers in a reaction volume of 20 μl. 2.5 μl of 1:5 diluted reverse transcription product was amplified using gene-specific primers and Power SYBR Green PCR master mix (Applied Biosystems, Carlsbad, CA) on a Roche Light Cycler 480. Results were calculated using ΔΔCT method and zebrafish ribosomal protein *L5b (rpl5b*) or human β*-actin* as a normalization control. The following primers were used: *cdh1*, forward 1 (5′-TCA GTA CAG ACC TCG ACC GGC CAA-3′) and reverse 1 (5′-AAA CAC CAG CAG AGA GTC GTA GG-3′) (22); *cdh1*, forward 2 (5′-GGC TTG TGT AAC AAC TGT GGG-3′) and reverse 2 (5′-GCC ACT GTG AAG GTG ATT TCG-3′); *cdh2*, forward (5′-TAG ACG CCG ATG GGA CAG TTA TGG-3′) and reverse (5′-CAG TAT CAC TGG CAC CTG TTT GGG-3′); *epcam*, forward (5′-AGA ACA TAA AGT GCG AGC CTG CGG-3′) and reverse and (5′-CTC AGT TTG GTG GCA TCA ATG GGC-3′); *rpl5b*, forward (5′-GGG GAT GAG TTC AAT GTG GAG-3′) and reverse (5′-CGA ACA CCT TAT TGC CAG TAG-3′ (35)); *zeb1a*, forward (5′-AGC AGA GGA GCA TCA GAG AAC GC-3′) and reverse (5′-GCA GTG CGG ACA GTT GTG CAG G-3′); *zeb1b*, forward (5′-TGA AAG AGG AGT GCG TGT CGG-3′) and reverse (5′-TGT AGC CAC GAG AGC AGT ACG-3′); β*-actin*, forward (5′-GCC CTG AGG CAC TCT TCC A-3′) and reverse (5′-TTG CGG ATG TCC ACG TCA-3′ (8)); *CDH1*, forward (5′-GTC CTG GGC AGA CTG AAT TT-3′) and reverse (5′-GAC CAA GAA ATG GAT CTG TGG-3′ (8)); *EPCAM*, forward (5′-ATG CCA GTG TAC TTC AGT TGG TGC-3′) and reverse (5′-GCC ATT CAT TTC TGC CTT CAT CAC C-3′); *ZEB1*, forward (5′-AAG AAT TCA CAG TGG AGA GAA GCC A-3′) and reverse (5′-CGT TTC TTG CAG TTT GGG CAT T-3′ (8)).

##### miRs

miR qRT-PCR analysis were carried out using Taqman miR assays (Applied Biosystems) for zebrafish miR-141, miR-200b, and control miR-26a following manufacturer-recommended protocols. 10 ng of total RNA/15 μl of reaction volume was used during reverse transcription. 2.5 μl of 1:3- diluted reverse transcription product was used for subsequent real-time PCR reactions performed on a Roche Light Cycler 480.

##### Western Blot

Embryos at 80% epiboly stage were enzymatically dechorionated, deyolked, and homogenized in triple detergent buffer (50 mm Tris-HCl (pH 8), 150 mm NaCl, 0.02% (w/v) NaN_3_, 0.5% (w/v) sodium deoxycholate, 0.1% (w/v) SDS, 1% (v/v) Nonidet P-40) for 40 min at 4 °C. 20 μg of total protein, estimated by the Bradford assay (Bio-Rad), were loaded onto a 10% polyacrylamide gel and transferred to a nitrocellulose membrane. The following primary antibodies were used: anti-E-cadherin antibody (1:5000; BD Transduction Laboratories^TM^, #610182), anti-β-actin antibody (1:5000; Sigma, #A5441).

##### Quantification of Gastrulation Movement Defects

Epiboly progress was quantified as position of vegetal blastoderm margin as percent of animal-vegetal distance. Deep cell layer thinning was quantified as thickness of deep cell layer at the animal pole as percentage of animal-vegetal extent of blastoderm. Deep cell migration toward the animal pole was quantified by measuring the angle between the foremost prechordal cells and the animal pole. The GNU Image Manipulation Program (GIMP) was used for measurements. For categorical quantifications (internalization, convergence, and EVL phenotypes), representative embryos for each category (normal, affected) are shown in the figures.

##### Chromatin Immunoprecipitation (ChIP)

Cross-linking was performed with 1% formaldehyde and stopped by the addition of glycine to a final concentration of 0.125 m. Cells were lysed in Nonidet P-40 lysis buffer (0.5% Nonidet P-40, 85 mm KCl, 5 mm Hepes (pH 7.9) and protease inhibitors (Roche Applied Science)) and disrupted by Dounce homogenization. Nuclei were resuspended in nuclei lysis buffer (50 mm Tris-HCl (pH 8), 10 mm EDTA, 1% SDS, and protease inhibitors) and sonicated (10 × 30 s on/30 s off, Diagenode Bioruptor). Chromatin containing 80 μg of DNA per experimental condition was precleared with Dynabeads® protein A and G (Invitrogen, 1:1 mixture). Immunoprecipitation was performed overnight at 4 °C in immunoprecipitation buffer (20 mm Hepes (pH 8), 0.2 m NaCl, 2 mm EDTA, 0.1% sodium deoxycholate, 1% Triton X-100, 1 mg/ml BSA, protease inhibitors) with Zeb1 (5 μg of Santa Cruz H102 (sc-25388 X) or 2.5 μl of Sigma Prestige (HPA027524)) and normal rabbit IgG control (5 μg of Santa Cruz (sc-2345)) antibodies. Immune complexes were captured with 50 μl of Dynabeads® protein A and G (1:1). Beads were washed with washing buffer (1 m Hepes (pH 7.9), 0.5 m EDTA, 10% Nonidet P-40, 10% sodium deoxycholate, 8 m LiCl) and Tris-EDTA, and precipitates were eluted (50 mm Tris (pH 8.0), 10 mm EDTA, 1% SDS) for 15 min at 65 °C. After the addition of 100 mm NaCl and digestion with 100 μg/ml proteinase K, cross-links were reversed by overnight incubation at 65 °C. DNA was purified with a QIAquick PCR purification kit, PCR-amplified (used primers: *EPCAM*, forward (5′-GCC AGG TAA AAG CTC AAA GG-3′) and reverse (5′-GCG GGA ACT GGA TAG AGG A-3′); *GAPDH*, forward (5′-TAC TAG CGG TTT TAC GGG CG-3′) and reverse (5′-TCG AAC AGG AGG AGC AGA GAG CGA-3′)) (14) and analyzed on an agarose gel.

##### Cell Dissociation and Aggregation Assay

Cell dissociation and reaggregation was performed as described (22). Classification of cluster composition was performed by visual inspection.

##### Confocal Microscopy

Confocal images were made using Zeiss LSM-510 confocal microscopes (Carl Zeiss Micro Imaging, Jena). Confocal acquisition parameters: LD LCI Plan-Apochromat 25×/N.A. 0.8; pixel size, 0.5 μm × 0.5 μm ×1 μm; excitation laser wavelengths at 488 and 543 nm, emission filter, BP500–530 IR, BP 565–615 IR.

##### Statistical Analysis

Statistical analyses of the qRT-PCR data (Figs. 2*K*, 3*A*, 4*C*, 5, *D* and *G*, and 7, *B* and *F*), the epiboly movement data (Figs. 2, *F* and *G*, 3, *E* and *F*, 7*E*), the data on deep cell migration toward the animal pole (Fig. 2*J*), and the data of the width of *cdh1* and *cdh2* expression domains (Fig. 4, *B* and *G*) were performed with Microsoft Excel software. The raw data were processed to calculate the S.E. (as indicated by *error bars* in the figures). Statistical significances were evaluated by the two-tailed Student's *t* test. Categorical data (Figs. 2*I*, 5*B*, and 6*M*) were presented as stacked-column graphs using the Microsoft Excel software. Statistical significances were evaluated by the two-tailed Fisher's exact test. Fisher's exact test was performed using VassarStats: Website for Statistical Computation. In all figures *, *p* < 0.05; **, *p* < 0.01; ***, *p* < 0.001.

## RESULTS

### 

#### 

##### Spatial and Temporal Correlation of zeb1a, zeb1b, cdh1, and cdh2 Expression

Analyses of primary cancers and cancer cell lines from different entities revealed an inverse relationship of ZEB1 and E-cadherin (encoded by the *CDH1* gene) expression and a positive correlation between ZEB1 and N-cadherin (encoded by the *CDH2* gene) expression (6, 36–38). Using zebrafish as a model organism, we wanted to determine whether these regulatory relationships also control cell adhesion systems during vertebrate gastrula and segmentation stages.

**FIGURE 1.**
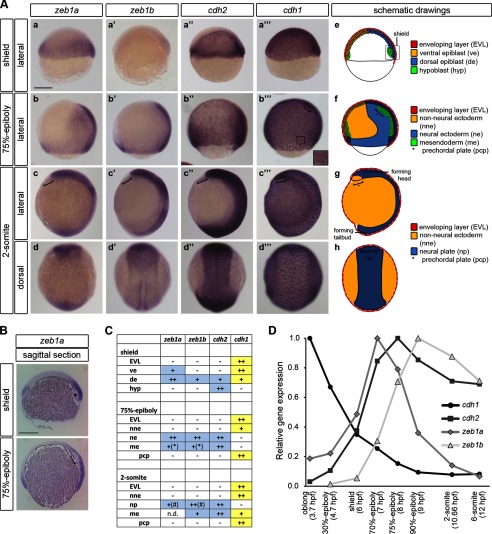
**Spatial and temporal correlation of *zeb1a*, *zeb1b*, *cdh1*, and *cdh2* expression.** *A*, shown are whole-mount ISH of WT embryos hybridized with either *zeb1a* (*a–d*), *zeb1b* (*a′–d′*), *cdh2* (*a′*–d′), or *cdh1* (*a‴–d‴*) antisense probe at the indicated developmental stages. The staining reaction time was adjusted for each stage, and therefore, stain intensities are not proportional to gene expression levels. *Brackets* in (*b‴*, *c–c‴*) indicate prechordal plate. The *inset* in (*b‴*) shows higher magnification of EVL cells, with *cdh1* transcripts prominent in perinuclear cytoplasm. In all lateral views dorsal is to the *right*. In all dorsal views animal pole is to the *top. Scale bar*, 200 μm. *e–h*, schematic drawings of zebrafish embryos at the indicated developmental stages are shown. In *e* a sagittal section is shown. In *f* an overlay of a sagittal section and a surface view is shown. *g* and *h* represent surface views. Orientation of the embryos is as in (*a–d‴*). *B*, shown are medial sagittal sections through whole embryos, hybridized with a probe against *zeb1a* at shield and 75%-epiboly. The *asterisk* indicates the involuting hypoblast. The *bracket* indicates the prechordal plate. *Scale bar*, 200 μm. *C*, the table summarizes the expression patterns of *zeb1a*, *zeb1b*, *cdh2*, and *cdh1. ve*, ventral epiblast; *de*, dorsal epiblast; *hyp*, hypoblast; *nne*, non-neural ectoderm; *ne*, neural ectoderm; *me*, mesendoderm; *pcp*, prechordal plate; *np*, neural plate, *n.d.*, not determined. Data were summarized from this figure and published work (15, 18, 39, 40). * and #, *zeb1a* and *zeb1b* are prominently expressed in the invaginating marginal mesendoderm cells (*) and in the anterior neural plate (#). *D*, transcript levels of *zeb1a*, *zeb1b*, *cdh1*, and *cdh2* during early WT embryo development (3.7–12 hpf) were determined by qRT-PCR. Expression was normalized to *rpl5b*. Expression levels of analyzed genes are calculated relative to the highest expression of each gene (set to 1) during analyzed time points.

Spatial expression analysis revealed that *cdh1* is mostly expressed in a complementary pattern to that of *cdh2*, *zeb1a,* and *zeb1b*. Briefly summarized, high *cdh1* transcript levels were detected in the non-neural ectoderm, the prechordal plate, and the EVL, where the *zeb* genes and *cdh2* were not expressed (15, 18, 39, 40) (Fig. 1, *A–C*). Analysis of temporal gene expression by qRT-PCR from late blastula to 6-somite stage showed that increasing expression of *zeb1* paralogs goes along with decreasing *cdh1* expression, whereas *cdh2* transcript levels increase in parallel with *zeb1a* and *zeb1b* (Fig. 1*D*). Therefore, correlations between *CDH1*, *CDH2*, and *ZEB1* genes that have been described in cancer may also exist in early zebrafish development.

##### zeb1a/b Double Knockdown Severely Affects Gastrulation

**FIGURE 2.**
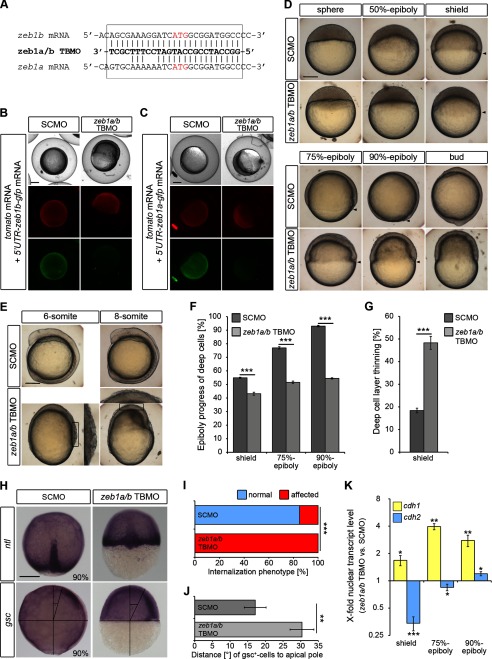
**Depletion of both *zeb1* paralogs (*zeb1a* and *zeb1b*) causes severe gastrulation defects.** *A*, sequence of *zeb1a/b* TBMO aligned with the translational start site region of *zeb1b* and *zeb1a* mRNA is shown. The start codons (ATG) are depicted in *red*. The *zeb1a/b* TBMO was designed against the initiation start site of *zeb1b* and has six nucleotides mismatched with the paralog *zeb1a. B* and *C*, MO specificity was demonstrated by knockdown of GFP reporter expression. Embryos were injected with *nls-tomato* mRNA (50 pg), *gfp-reporter* mRNA (50 pg) (*5′UTR-zeb1b-gfp mRNA* (*B*) and *5′UTR-zeb1a-gfp mRNA* (*C*)) and either SCMO (4 ng) or *zeb1a/b TBMO* (4 ng) and assayed for NLS-Tomato and GFP expression 8 h later. The fusion reporter construct *5′UTR-zeb1b-gfp* mRNA contains the *zeb1b* mRNA sequence depicted in the *box* in *A* fused to the *gfp* coding sequence. The fusion reporter construct *5′UTR-zeb1a-gfp* mRNA contains the *zeb1a* mRNA sequence depicted in the *box* in *A* fused to the *gfp* coding sequence. *zeb1a/b* morphants expressed NLS-Tomato comparable to control embryos but showed less expression of both GFP reporters, indicating that the *zeb1a/b* TBMO not only reduces translation of *zeb1b* but also efficiently binds to the translational start site region of *zeb1a. D* and *E*, live WT embryos were injected with SCMO or *zeb1a/b* TBMO at the indicated stages. Lateral views are shown, with the animal pole toward the top and dorsal to the *right. Arrowheads* indicate vegetal front of blastoderm. *Scale bar*, 200 μm. *F* and *G*, shown is quantification of epiboly progress and deep cell layer thinning in SCMO-injected embryos and *zeb1a/b* double morphants shown in *D* (shield, *n* = 14 embryos each; 75% epiboly, *n* = 13 embryos each; 90% epiboly, *n* = 15 embryos each). Values are the mean ± S.E. *H*, shown is whole-mount ISH of SCMO-injected embryos and *zeb1a/b* double morphants. Embryos were hybridized with *no tail* (*ntl*) (*upper panel*) antisense probe to evaluate the internalization movement of axial mesodermal cells or *goosecoid* (*gsc*) (*lower panel*) antisense probe to evaluate the migration of prechordal cells toward the animal pole. Dorsal views (*upper panels*) are shown with the animal pole to the *top*. Lateral views (*lower panels*) with are shown with the dorsal to the *right*. The angle between the foremost prechordal cells and the animal pole is depicted in the *lower panels. Scale bar*, 200 μm. *I*, quantification of the internalization phenotype (*n* = 20 embryos each) shown in *H. J*, shown is quantification of the deficit in prechordal mesoderm migration toward the animal pole (*n* = 15 embryos each) shown in *H*. Values are the mean ± S.E. *K*, shown is the time series qRT-PCR data of *cdh1* mRNA (*yellow*) and *cdh2* mRNA (*blue*) expression in nuclear extracts of *zeb1a/b* double morphants relative to SCMO-injected embryos (shield, *n* = 6; 75% epiboly, *n* = 4; 90% epiboly, *n* = 4). Expression values were normalized to *rpl5b. cdh1* and *cdh2* expression in SCMO-injected embryos was set to 1. Values are the mean ± S.E.

To determine whether Zeb1a and Zeb1b affect *cdh1* and *cdh2* gene expression during early zebrafish development, we performed knockdown of both *zeb1a* and *zeb1b* transcripts using a TBMO that efficiently inhibits the translation of both *zeb1* paralogs (Fig. 2, *A–C*). *zeb1a/b* morphants exhibited two prominent phenotypes. First, gastrulation was delayed and did not progress normally (Fig. 2*D*). Second, the surface of the EVL appeared rough, and detachment of superficial cells was observed (Fig. 2*E*), a phenotype also described in a recent study (16). Morphants died during early somitogenesis stages when embryos dissociated. The observed delay of epiboly movements upon *zeb1a/b* knockdown (Fig. 2*D*) was highly significant when quantified by measuring epiboly progress and deep cell layer thinning (Fig. 2, *F* and *G*). Analysis of *no tail* (*ntl*)-expressing chordamesoderm cells revealed that internalization did occur, but *ntl*-expressing cells extended less in animal direction in all analyzed *zeb1a/b* morphant embryos (Fig. 2, *H*, *upper panel*, and *I*). Expression analysis of *goosecoid* (*gsc*)-expressing prechordal mesoderm cells revealed impaired migration in *zeb1a/b* morphants relative to controls (Fig. 2, *H*, *lower panel*, and *J*). Injection of MOs that selectively inhibit either *zeb1a* (*zeb1a* TBMO) or *zeb1b* (*zeb1b* SBMO) resulted in milder phenotypes. A combination of both paralog-specific MOs produced an additive effect when compared with single *zeb1b* knockdown (data not shown), arguing for partially redundant activities of both paralogs.

##### Zeb1b Controls Epiboly Progression by Repressing cdh1

Given the prominent role of E-cadherin in zebrafish gastrulation, we determined the effects of Zeb1 on E-cadherin expression. Because large amounts of *cdh1* transcripts are maternally deposited into the cytoplasm (26), the transcriptional effects of Zeb1 on zygotic *cdh1* expression should best be determined from the amount of new *cdh1* transcripts in the nuclei after mid-blastula transition (three hpf in zebrafish). Therefore, nuclear RNA extracts were prepared and analyzed by qRT-PCR. Expression of zygotic *cdh1* was increased in *zeb1a/b* morphants throughout gastrulation, whereas *cdh2* transcript levels were considerably decreased only at shield stage (Fig. 2*K*). The elevated *cdh1* levels in *zeb1a/b* morphants may in part be responsible for the gastrulation phenotype. Therefore, by applying a low *cdh1* MO dose, we tested whether a slight reduction of *cdh1* levels would partially rescue the gastrulation phenotype of *zeb1a/b* morphants. Indeed, combined knockdown of *cdh1* and *zeb1a/b* could partially rescue the epiboly and emboly defects of *zeb1a/b* morphants (data not shown; embryos, however, did not complete somitogenesis). Taken together, these data suggest that Zeb1 may affect gastrulation at least in part through regulation of E-cadherin.

**FIGURE 3.**
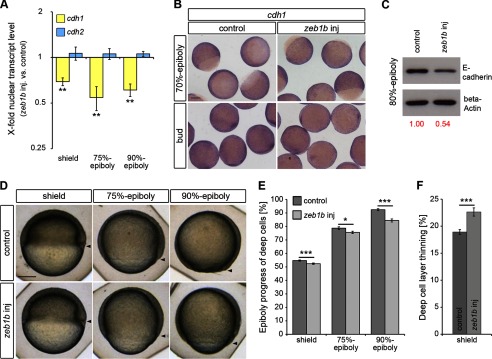
**Zeb1b controls E-cadherin expression and epiboly progression.** *A*, shown is the time series qRT-PCR data of *cdh1* mRNA (*yellow*) and *cdh2* mRNA (*blue*) expression in nuclear extracts of *zeb1b*-overexpressing embryos relative to control embryos (*n* = 3 each for all stages). Expression values were normalized to *rpl5b. cdh1* and *cdh2* expression in control embryos was set to 1. Values are the mean ± S.E. *B*, shown is whole-mount ISH of control and *zeb1b*-overexpressing embryos. Embryos were hybridized with a *cdh1* antisense probe at the indicated developmental stages. *Scale bar*, 200 μm. *C*, shown is a Western blot of E-cadherin (*top*) and β-actin (*bottom*, loading control) from 80% epiboly control and *zeb1b*-overexpressing embryos. Only the larger isoform of E-cadherin, which is dominant during gastrulation (34), was detected by the antibody. Relative protein expression was quantified using *Image J* software. *D*, shown are live control and *zeb1b*-overexpressing embryos at the indicated stages. Lateral views are shown, with the vegetal pole toward the *bottom* and dorsal to the *right. Arrowheads* indicate the vegetal front of blastoderm. *Scale bar*, 200 μm. *E* and *F*, shown is quantification of epiboly progress and deep cell layer thinning in control embryos and *zeb1b*-overexpressing embryos shown in *D* (shield, *n* = 40 embryos each; 75% epiboly, *n* = 32 embryos each; 90% epiboly, *n* = 37 embryos each). Values are the mean ± S.E.

For gain-of-function studies we focused on Zeb1b, which has a higher sequence similarity to human ZEB1 than Zeb1a, especially in the zinc fingers and the homeodomain (data not shown). Zygotic *cdh1* transcript levels were decreased in cell nuclei of *zeb1b*-overexpressing embryos at shield, 75% and 90% epiboly stages, whereas *cdh2* transcript levels were unaffected (Fig. 3*A*). Whole-mount ISH confirmed reduced levels of *cdh1* expression in *zeb1b*-overexpressing embryos at bud stage (Fig. 3*B*). However, at 70% epiboly the ISH technique could not detect a change in *cdh1* transcript levels upon *zeb1b* overexpression, which is likely caused by the fact that the ISH technique is not sensitive enough to reveal Zeb1b-induced changes of zygotically transcribed *cdh1* mRNA levels against the background of the still persistent large amount of maternal-derived *cdh1* mRNA at this stage. Western blot analysis confirmed that *zeb1b*-overexpressing embryos have lower levels of E-cadherin protein (Fig. 3*C*, see also Goudarzi *et al.* (41)). To analyze whether *zeb1b* overexpression affects epiboly movements, we measured epiboly progress at shield, 75% and 90% epiboly stages, and deep cell layer thinning at shield stage. Both measurements revealed that epiboly is significantly delayed in *zeb1b*-overexpressing embryos (Fig. 3, *D–F*). Thus, Zeb1 activity may contribute to control of epiboly by repression of *cdh1* but does not appear to affect *cdh2.*

##### Zeb1b Affects Convergence Movements

We investigated for two reasons the role of Zeb1b during segmentation stages, when neurulation occurs in zebrafish. First, neurulation is characterized by an E- to N-cadherin switch with E-cadherin being down-regulated in the neural ectoderm and retained in the non-neural ectoderm, whereas N-cadherin is up-regulated in the *zeb1b*-expressing neural plate (Fig. 1). Second, a previous study indicated that Zeb1b acts downstream of the neural inducers Noggin and Chordin and that overexpression of Zeb1b results in an expansion of the neuroectoderm (15).

**FIGURE 4.**
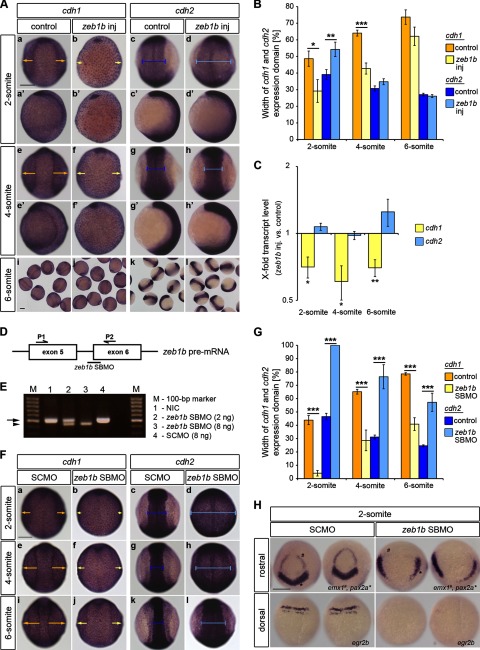
**Zeb1b overexpression and knockdown affect convergence movements.** *A*, shown is whole-mount ISH of control and *zeb1b*-overexpressing embryos. Embryos were hybridized with either a *cdh1* (*a–b′*, *e–f′*, *i*, and *j*) or *cdh2* (*c–d′*, *g–h′*, *k*, and *l*) antisense probe at the indicated developmental stages. In all dorsal views (*a–h*) animal pole is to the *top*. In all lateral views (*a′–h′*) dorsal is to the *right. Yellow arrows* indicate the width of the *cdh1* expression domain, corresponding to the non-neural ectoderm. *Blue bars* indicate the width of the *cdh2* expression domain, corresponding to the neural ectoderm. *Scale bar*, 200 μm. *B*, shown is quantification of the width of *cdh1* (*yellow*) and *cdh2* (*blue*) expression domains (2-somite, *n* = 13 embryos each for *cdh1* and *n* = 14 embryos each for *cdh2*; 4-somite, *n* = 47 embryos each for *cdh1* and *n* = 43 embryos each for *cdh2*; 6-somite, *n* = 17 embryos each for *cdh1* and *n* = 16 embryos each for *cdh2*). Values are the mean ± S.E. *C*, shown is the time series qRT-PCR data of *cdh1* mRNA (*yellow*) and *cdh2* mRNA (*blue*) expression in *zeb1b*-overexpressing embryos relative to control embryos (*n* = 4 each for all stages). Expression values were normalized to *rpl5b. cdh1* and *cdh2* expression in control embryos was set to 1. Values are the mean ± S.E. *D*, shown is a schematic illustration of the *zeb1b* pre-mRNA exon5–6 region and the location of the *zeb1b*-specific SBMO (*black bar*; *zeb1b* SBMO) as well as the positions of primers used for the RT-PCR reactions (*black half-arrows*; *P1* and *P2*). *E*, the agarose gel shows PCR products from various conditions. *Lane 1*, shown is the RT template from non-injected control embryos (*NIC*). *Lane 2*, shown is the RT template from embryos injected with 2 ng of *zeb1b* SBMO. *Lane 3*, shown is the RT template from embryos injected with 8 ng *zeb1b* SBMO. *Lane 4*, shown is the RT template from embryos injected with 8 ng of SCMO. The *black arrow* indicates the expected WT PCR product of 505 bp. The *arrowhead* indicates the shorter PCR product appearing after *zeb1b* SBMO injection. Template cDNA was generated from 75% epiboly stage embryos. The *zeb1b* SBMO (intron 5 exon 6; i5e6) targets the splice acceptor site of exon 6. Based on sequencing of amplified cDNA *zeb1b* SBMO leads to the deletion of the first 60 nucleotides of exon 6 that partially code for the fourth zinc finger of Zeb1b. *F*, shown is whole-mount ISH of SCMO- and *zeb1b* SBMO-injected embryos. Embryos were hybridized with either a *cdh1* (*a*, *b*, *e*, *f*, *i*, and *j*) or *cdh2* (*c*, *d*, *g*, *h*, *k*, and *l*) antisense probe at the indicated developmental stages. Dorsal views are shown with the animal pole to the *top. Yellow arrows* indicate the width of the *cdh1* expression domain, corresponding to the non-neural ectoderm. *Blue bars* indicate the width of the *cdh2* expression domain, corresponding to the neural ectoderm. *Scale bar*, 200 μm. *G*, shown is quantification of the width of *cdh1* (*yellow*) and *cdh2* (*blue*) expression domains (2-somite, *n* = 8 embryos (SCMO) and *n* = 7 embryos (*zeb1b* SBMO) for *cdh1* and *n* = 12 embryos (SCMO) and *n* = 10 embryos (*zeb1b* SBMO) for *cdh2*; 4-somite, *n* = 11 embryos (SCMO) and *n* = 5 embryos (*zeb1b* SBMO) for *cdh1* and *n* = 10 embryos (SCMO) and *n* = 7 embryos (*zeb1b* SBMO) for *cdh2*; 6-somite, *n* = 10 embryos (SCMO) and *n* = 8 embryos (*zeb1b* SBMO) for *cdh1* and *n* = 10 embryos (SCMO) and *n* = 7 embryos (*zeb1b* SBMO) for *cdh2*). Values are the mean ± S.E. *H*, shown is whole-mount ISH of SCMO-injected embryos and *zeb1b* morphants. Embryos were hybridized with *emx1* and *pax2a* (*upper panel*) and *egr2b* (*lower panel*) antisense probe. Rostral views (*upper panels*) are shown with the ventral toward the *top*. Dorsal views (*lower panels*) are shown with the animal pole toward the *top. Scale bar*, 200 μm.

To address whether Zeb1b is crucial for the change of cadherin expression during zebrafish neurulation, we analyzed the expression patterns and transcript levels of *cdh1* and *cdh2* in *zeb1b*-overexpressing embryos during early segmentation. The width of *cdh1* and *cdh2* expression domains, representing the non-neural ectoderm and neural plate, respectively, was measured after orienting embryos in a dorsal view (Fig. 4*A*, *a–d* and *e–h*). The *cdh1* expression domain (*yellow arrows*) was significantly smaller in *zeb1b* mRNA-injected embryos at the 2- and 4-somite stage, whereas the *cdh2* expression domain (*blue bars*) was only significantly wider at the 2-somite stage compared with controls. At the six-somite stage, no significant differences could be detected (Fig. 4*A*, *i–l*, and *B*). Thus, Zeb1b overexpression transiently shifted the boundary between neural and non-neural ectoderm in favor of neural ectoderm during early segmentation stages. Next, we investigated *cdh1* and *cdh2* transcript levels by qRT-PCR. We found a significant decrease of *cdh1* transcript levels in *zeb1b*-overexpressing embryos at all analyzed segmentation stages. However, a difference in *cdh2* transcript levels at the corresponding stages was not detected (Fig. 4*C*). Therefore, we conclude that the transient expansion of the *cdh2*-expressing neural plate is, rather, a consequence of decreased *cdh1* expression that results in defects in convergence movements (21) than a consequence of a boosted cadherin switching.

For loss-of-function studies we focused on *zeb1b* morphants, as *zeb1a/b* morphants could not be analyzed during segmentation stages due to embryo dissociation (Fig. 2*E*). Depletion of *zeb1b* using a SBMO (Fig. 4, *D* and *E*) led to an expansion of the *cdh2*-expressing neural plate at the expense of the *cdh1*-expressing non-neural ectoderm at all analyzed segmentation stages (Fig. 4, *F* and *G*). Analysis of *cdh1* and *cdh2* transcript levels by qRT-PCR at the four-somite stage revealed an unchanged expression of *cdh1* and an increased expression of *cdh2* (data not shown). Unchanged *cdh1* expression may be due to the expression of *zeb1a* that is not affected by the *zeb1b* SBMO. The unexpected increase of *cdh2* expression may either be a secondary effect of the altered morphogenesis of the neural plate, which leads to its strong expansion, or due to an increased amount of neural tissue. To distinguish between these possibilities, we examined the expression of early regional neural markers. Expression domains of forebrain (*emx1*), midbrain (*pax2a*), and hindbrain (*egr2b*) markers were laterally extended, but their expression levels were reduced or even absent upon Zeb1b knockdown (Fig. 4*H*). The above defects are likely not caused by a general developmental delay in *zeb1b* morphants, as two markers of early territories (*ntl* and *gsc*) were normally expressed in morphants (data not shown). Additionally, reduced expression of neural markers in *zeb1b* morphants is consistent with previously published data describing Zeb1b as neural inducer (15).

Taken together, these data show that Zeb1b contributes to regulation of convergence movements. Because *cdh1* levels were not changed in *zeb1b* morphants, E-cadherin-mediated cell-cell adhesion does not seem to be the sole mediator of the Zeb1-dependent convergence defects. Furthermore, our data are consistent with the notion that Zeb1b may promote neuroectodermal development. However Zeb1b is not responsible for the E- to N-cadherin switch during neurulation.

##### EPCAM Is a Transcriptional Target of ZEB1

**FIGURE 5.**
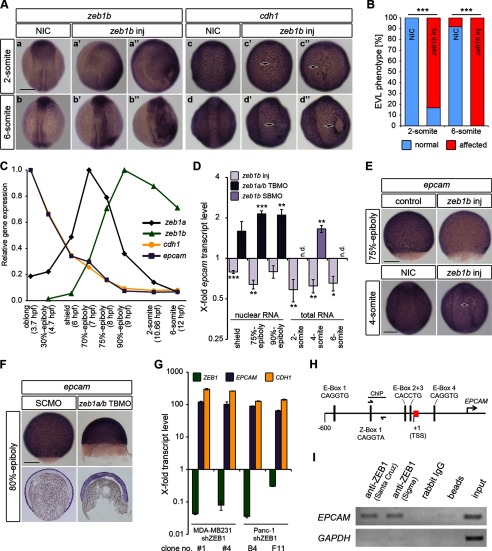
**Human ZEB1 and the zebrafish paralogs Zeb1a and Zeb1b control *EPCAM* (*epcam*) expression.** *A*, shown is whole-mount ISH of non-injected control embryos (*NIC*) and *zeb1b*-overexpressing embryos (*zeb1b inj*). RNA was injected into one blastomere of 4-cell stage embryos. The amount of injected *zeb1b* mRNA per cell was 4-fold higher as compared with Fig. 4*A*. Embryos were hybridized with either a *zeb1b* (*a–b′*) or *cdh1* (*c–d′*) antisense probe at the indicated developmental stages. All embryos are orientated in a dorsal view with the animal pole toward the *top. Black arrows* indicate affected EVL integrity (quantified in *B*). *Scale bar*, 200 μm. *B*, shown is quantitative analysis of the EVL phenotype shown in *A* (*n* = 12 embryos each). *C*, transcript levels of *zeb1a* (*black*), *zeb1b* (*green*), *cdh1* (*yellow*), and *epcam* (*purple*) during early WT embryo development (3.7–12 hpf) were quantified by qRT-PCR. Expression was normalized to *rpl5b*. Expression levels of analyzed genes are presented relative to the highest expression of each gene (set to1) during analyzed time points. *D*, shown is a time series qRT-PCR data of nuclear or total *epcam* mRNA expression in *zeb1b*-overexpressing embryos, *zeb1a/b* morphants, and *zeb1b* morphants relative to control embryos (*n* = 3–6 per condition). Expression values were normalized to *rpl5b. epcam* expression in control embryos was set to 1. Values are the mean ± S.E.; *n.d.*, not determined. *E*, whole-mount ISH of control and *zeb1b*-overexpressing embryos is shown. In the *upper panel* embryos were injected with control *gfp* mRNA or *zeb1b* mRNA at the one-cell stage. In the *lower panel* non-injected control embryos and *zeb1b*-overexpressing embryos (*zeb1b inj*), where *zeb1b* RNA was injected into one blastomere of 4-cell stage embryos, are depicted. Embryos were hybridized with an *epcam* antisense probe at the indicated stages. Dorsal views are shown with the animal pole toward the *top. Scale bar*, 200 μm. *F*, shown is whole-mount ISH (*upper panel*) and sagittal sections through stained embryos (*lower panel*) injected with SCMO or *zeb1a/b* TBMO and hybridized with a probe against *epcam* at 80%-epiboly. *Scale bar*, 200 μm. *G*, shown are transcript levels of *ZEB1* (*green*), *EPCAM* (*purple*), and *CDH1* (*yellow*) in characteristic short hairpin control (shGFP), and shZEB1 knockdown clones of human breast (MDA-MB231) and human pancreatic (Panc-1) cancer cell lines were quantified by qRT-PCR. Expression was normalized to β*-actin. ZEB1*, *EPCAM*, and *CDH1* mRNA expression is relative to control clone MDA-MB231 shGFP #1 and Panc-1 shGFP D4. Expression in control clones was set to 1. Values represent the mean ± S.E. of technical triplicates. *H*, shown is a schematic representation of the putative promoter of human *EPCAM* on chromosome 2p21. The sequence-predicted ZEB1 binding sites (E-boxes 1–4 and Z-box 1) and the region amplified for ChIP are indicated. Primers used for ChIP analysis are shown as *half-arrows*. All numbers are in bp relative to the transcription start site (*TSS*) of *EPCAM. I*, ChIP shows *in vivo* binding of ZEB1 to the putative promoter of human *EPCAM*. Lysates from MDA-MB231 cells were subjected to ChIP by two different anti-ZEB1 antibodies (from Santa Cruz or Sigma). Rabbit IgG and a chromatin sample without the addition of antibody (beads) were used as negative controls. 5% of the supernatant of the antibody isotype control after immunoprecipitation was used as the input control. Eluted DNA was subjected to PCR for *EPCAM* promoter. *GAPDH* promoter was used as negative control.

To better understand the impact of Zeb1b on embryos during early segmentation stages, we generated mosaic embryos in which Zeb1b was overexpressed in a subpopulation of cells by co-injecting *zeb1b* mRNA and *gfp* mRNA into a single blastomere at the four-cell stage. Embryos with mosaic GFP expression were further analyzed by whole-mount ISH (Fig. 5*A*). *zeb1b* transcripts were found to be overexpressed in a mosaic manner, and some embryos displayed a curved developing spine (Fig. 5*A* (*a*′ and *b*′)). Spatial analysis of *cdh1* expression confirmed that Zeb1b negatively regulates *cdh1* expression in ectodermal cells with epidermal fate. In addition, Zeb1b overexpression led to strongly reduced expression of *cdh1* in individual EVL cells and local disruption of epithelial EVL integrity in most of the embryos (Fig. 5*A* (*arrows* in *c*′ and *d*′)). The decreased E-cadherin level in *zeb1b*-overexpressing embryos is unlikely the sole cause of the severe EVL phenotype, as *cdh1* morphants and mutants develop a normal EVL (21). Also, combined *zeb1b* overexpression and *cdh1* depletion could only partially and transiently improve the *zeb1a/b* morphant phenotype (data not shown). Recently, it has been shown that E-cadherin and Epcam, which are both highly expressed in EVL cells, are required in a partially redundant fashion to establish EVL epithelial integrity (30). Given that relatively large amounts of *epcam* transcripts are maternally deposited (30, 42) (Fig. 5*C*), we measured the expression level of zygotic *epcam* transcripts from isolated nuclei during gastrulation and from total RNA during segmentation stages. *epcam* transcript levels were decreased in *zeb1b*-overexpressing embryos and increased in *zeb1a/b* and *zeb1b* morphants at the stages analyzed (Fig. 5*D*). Whole-mount ISH of 75% epiboly-stage embryos confirmed reduced levels of *epcam* expression in *zeb1b*-overexpressing embryos (Fig. 5*E*, *upper panel*). Furthermore, mosaic embryos in which Zeb1b was overexpressed in a subpopulation of cells displayed regional lower *epcam* expression, convergence movement defects, and severely affected EVL integrity (Fig. 5*E*, *lower panel*). More interestingly, however, we found that depletion of both *zeb1* paralogs led to an ectopic *epcam* expression in the deep cells, where it is normally not expressed (Fig. 5*F*). Thus, Zeb1a/b repress *epcam* expression in deep cells during normal development. The combined effects of Zeb1b overexpression or Zeb1a and/or Zeb1b knockdown on *cdh1* and *epcam* transcription may at least in part explain the gastrulation movements and EVL integrity defects. Next we investigated whether the negative correlation between Zeb1 paralogs and *epcam* expression observed in zebrafish also holds true in cellular cancer models. We compared *EPCAM* mRNA levels in MDA-MB231 breast and Panc-1 pancreatic cancer cell clones with stable short hairpin RNA-mediated knockdown of ZEB1 (shZEB1 clones) to that in control knockdown (shGFP) clones (14). In both human cancer cell lines knockdown of ZEB1 resulted in elevated *EPCAM* and *CDH1* expression (Fig. 5*G*). By ChIP with chromatin from MDA-MB231 breast cancer cells, we could show that endogenous ZEB1 binds to the native promoter region of *EPCAM* that has five putative binding sites (E-boxes 1–4 and Z-Box-1) for ZEB1. One of those is restricted to ZEB factors (Z-Box 1; Fig. 5, *H* and *I*). The four remaining are perfect E-boxes, which may bind ZEB factors and other EMT activators like Snail (43). These data indicate that the transcriptional repressor ZEB1 can directly suppress expression of *EPCAM* by binding to its putative promoter.

##### Zeb1 Controls Cell Adhesion

**FIGURE 6.**
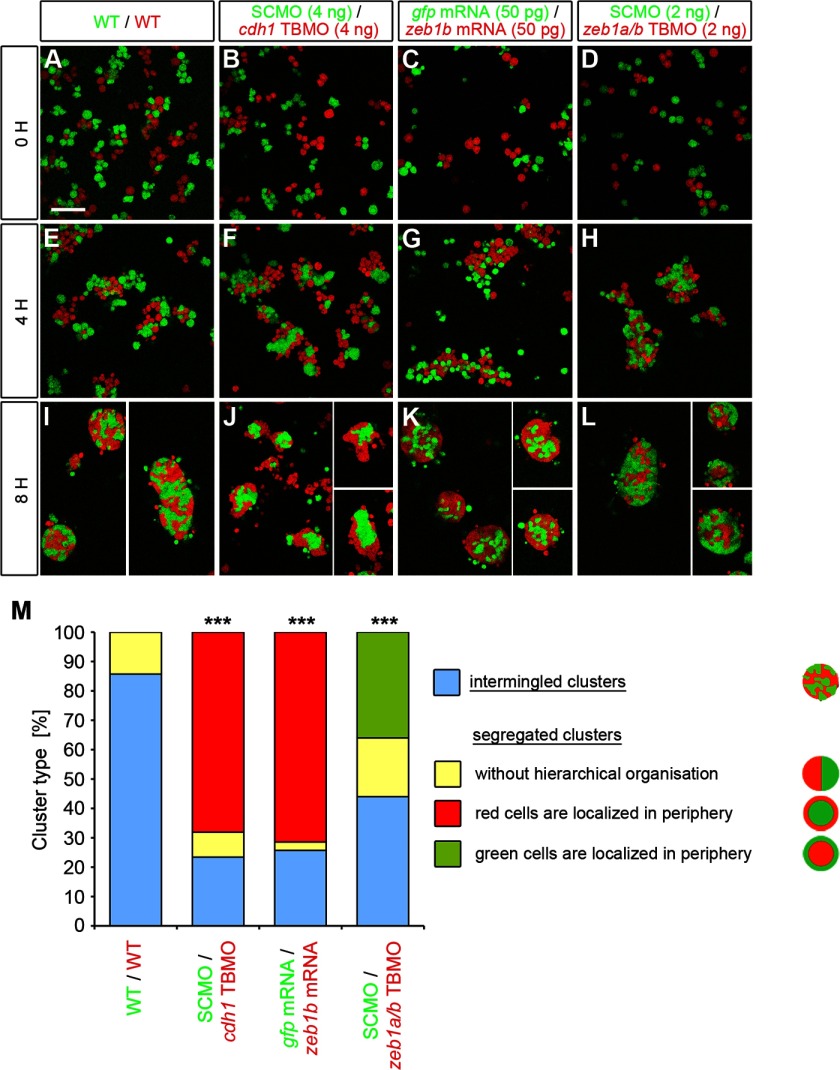
**Zeb1 controls blastoderm cell cohesiveness.** *A—L*, embryos were microinjected at the one-cell stage with mRNA or MOs as indicated at the *top of the panels* together with either Alexa488-dextran (*green*) or rhodamine-dextran (*red*) were mixed at sphere stage in equal proportions *in vitro* and dissociated. Primary co-cultures of those sphere stage embryo cells were plated on fibronectin-coated dishes and allowed to re-aggregate for 8 h. Proper dissociation was controlled within the first hour after plating (*A–D*). Four hours after plating, cells began to form clusters (*E–H*). Cell clusters were imaged after 8 h of incubation (*I–L*) and categorized as intermingled or separated. *Scale bar*, 100 μm. *M*, shown are measurements of the percentage of intermingled and segregated clusters. Segregated clusters were further classified in clusters without hierarchical organization of green and red cells (*yellow*), clusters where the red cells surround the green cells (*red*), and clusters where the green cells surround the red cells (*green*). *p* values are *versus* WT/WT control. Number of analyzed clusters are *n* = 35 for WT/WT, *n* = 47 for SCMO/*cdh1* TBMO, *n* = 35 for *gfp* mRNA/*zeb1b* mRNA, and *n* = 50 for SCMO/*zeb1a/b* TBMO.

Relative adhesion strength may be evaluated in cell reaggregation experiments, in which cells with lower adhesiveness are localized to the periphery of clusters, whereas more adherent cells are localized centrally (44). To evaluate cell adhesion in *zeb1b-*overexpressing and *zeb1a/b* morphant embryos, we carried out cell dissociation and reaggregation assays using zebrafish embryonic cells *ex vivo*. Color-labeled dissociated cells of sphere stage control and experimental embryos were mixed and plated in combinations (Fig. 6, *A–L*). After 8 h of incubation, reaggregated cell clusters were categorized as intermingled (aggregates of randomly mixed cells) or segregated (aggregates where green and red cells sorted out into distinct territories). As a control for intermingled clusters, co-cultures of differentially labeled dissociated WT embryos were performed (Fig. 6, *A*, *E*, and *I*).

Shortly after dissociation, differently labeled plated cells appear efficiently mixed (Fig. 6, *A–D*). After four hours of incubation, intermingled clusters started to form (Fig. 6, *E–H*). After 8 h of incubation, the WT/WT co-cultures formed mostly intermingled clusters (Fig. 6, *I* and *M*). SCMO/*cdh1* TBMO and *gfp* mRNA/*zeb1b* mRNA co-cultures predominantly formed segregated clusters where the green control cells were found centrally (Fig. 6, *J*, *K*, and *M*). To analyze cell-cell adhesion in *zeb1a/b* morphants, we used a low dose of 2 ng of *zeb1a/b* TBMO. Under these conditions clusters were formed after 8 h of incubation, and nearly 60% of those were segregated. In nearly two-thirds of those segregated clusters, the red *zeb1a/b*-deficient cells were localized in the center of the cluster (Fig. 6, *L* and *M*). These data demonstrate that reduction of Zeb1a/b activity significantly affects cell adhesion, and the central location of knockdown cells suggests higher adhesion levels. Co-cultures of cells from experimental embryos injected with high dose *zeb1a/b* TBMO (4 ng) did not form mixed clusters after 8 h of incubation. Although control cells adhered to each other, *zeb1a/b*-deficient cells were not able to efficiently adhere to each other or to control cells (data not shown). This finding is in line with the phenotype of *zeb1a/b* morphants at the beginning of segmentation, when embryos showed a rough surface, probably due to severely affected cell-cell adhesion (Fig. 2*E*). Our results indicate that modulation of E-cadherin and Epcam expression by Zeb1a and Zeb1b controls cell-cell adhesion of zebrafish blastoderm cells.

##### The Regulatory Feedback Loop of Zeb1 and miR-200 Is Functional but Has Only Minor Impact on Zebrafish Gastrulation

**FIGURE 7.**
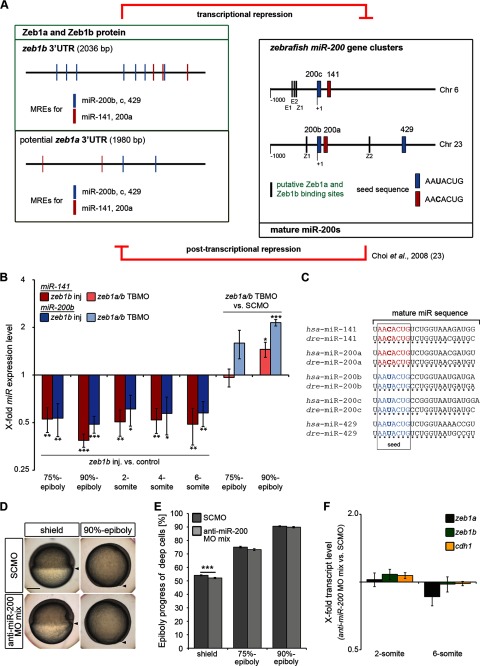
**Analysis of the potential reciprocal Zeb1a/b-miR-200 negative feedback loop.** *A*, shown is a schematic representation of the reciprocal Zeb1a/b-miR-200 feedback loop. *Left side*, shown is a scheme of the genomic organization of the *zeb1b* 3′UTR and the putative *zeb1a* 3′-UTR with their miR-200 family MREs. MREs were identified with the program RNAhybrid, which finds the energetically most favorable hybridization sites of a short RNA (to which miRs belong) in a long RNA (3′-UTR) (66). The minimum free energy of hybridization was set to <−14 kcal/mol. *Right side*, shown is a scheme of the genomic organization of the miR-200c-141 and miR-200b-a-429 clusters on zebrafish chromosome 6 and 23, respectively. E-(E) and Z-boxes (Z) as putative Zeb1a and Zeb1b binding sites are indicated. *B*, shown is a time series qRT-PCR data of miR-141 and miR-200b expression in *zeb1b*-overexpressing embryos (*left side*; injected with 100 pg *zeb1b* mRNA and 30 pg *gfp* mRNA) or *zeb1a/b* double morphants (*right side*; injected with 4 ng *zeb1a/b* TBMO) relative to control embryos (*left side*; injected with 130 pg *gfp* mRNA; *right side*; injected with 4 ng SCMO). Expression values were normalized to miR-26a. miR-141 and miR-200b expression in control embryos was set to 1 (*n* = 4 per condition; values represent the mean ± S.E.). *C*, a comparative genomic analysis of the miR-200 family members in human (*hsa*) and zebrafish (*dre*) indicates extensive conservation with respect to the mature miR and the seed sequences. *D*, shown are live control and miR-200 family knockdown embryos at the indicated stages. Lateral views are dorsal to the *right. Scale bar*, 200 μm. *E*, shown is quantification of epiboly progress in SCMO-injected embryos and miR-200 family-deficient embryos shown in *D* (shield, *n* = 25 embryos each; 75%-epiboly, *n* = 34 embryos each; 90% epiboly, *n* = 33 embryos each). Values are the mean ± S.E. *F*, shown are qRT-PCR data of *zeb1a*, *zeb1b*, and *cdh1* mRNA expression in miR-200 family-deficient embryos relative to SCMO-injected control embryos at 2- and 6-somite stage (*n* = 5 per condition). Expression values were normalized to *rpl5b. zeb1a*, *zeb1b*, and *cdh1* expression in control embryos was set to 1. Values are mean ± S.E.

Studies in human cancer cell lines revealed that ZEB1 and the miR-200 family are linked in a reciprocal negative feedback loop (13, 14). Zebrafish miR-200a and miR-200b have similar but not identical seed sequences and are together sufficient to post-transcriptionally repress Zeb1b expression by binding to their miR response elements (MREs) in the *zeb1b* 3′-UTR (23) (Fig. 7*A*). However, transcriptional repression of the miR-200 family members by Zeb1 has not been investigated in zebrafish so far. We measured the expression of miR-141 and miR-200b, located in the two different miR-200 family clusters, in *zeb1b*-overexpressing embryos and *zeb1a/b* morphants by qRT-PCR. Expression of miR-141 and -200b was significantly decreased in *zeb1b*-overexpressing embryos compared with control siblings during gastrulation and early segmentation. Analysis of *zeb1a/b* morphants revealed a significantly increased expression of both miRs at 90% epiboly stage but not earlier during gastrulation (Fig. 7*B*).

We also performed *in silico* analyses of the zebrafish miR-200 clusters and the 3′-UTRs of *zeb1a* and *zeb1b* (Fig. 7*A*). miR-200c and miR-141 map closely on chromosome 6, and the stem-loop sequences are separated by a 118-base pair spacer sequence. This spacer and the putative promoter 1 kb upstream from the miR-200c stem-loop contain three potential binding sequences for Zeb1a and Zeb1b (marked E1, E2, and Z1). The miR-200b-a-429 cluster is located on zebrafish chromosome 23. The stem-loop sequences of miR-200b and miR-200a are separated only by a 49-base pair spacer sequence, whereas the spacer between miR-200a and miR-429 comprises 1569 base pairs. These spacers and the putative promoter 1 kb upstream of the miR-200c stem-loop contain two potential binding sequences for Zeb1a and Zeb1b (marked Z1 and Z2). It was previously shown that the overall miR gene structure of both clusters, including the seed sequences, is highly conserved from zebrafish to mouse (23), and the Z-box and two E-boxes of the miR-200c-141-cluster are conserved between zebrafish and human (14). Alignments of human (*hsa*) and zebrafish (*dre*) mature miR sequences also revealed high conservation (Fig. 7*C*). We analyzed the 3′-UTRs of *zeb1a* and *zeb1b* for potential MREs for the miR-200 family (Fig. 7*A*, *left side*). The *zeb1a* 3′UTR contains 4, and the *zeb1b* 3′UTR 10 miR-200 family MREs. To investigate their functional relevance, we generated miR-200 morphants by injection of a triple anti-miR-200 MO mix (miR-141 MO, miR-200b MO, and miR-429 MO) that was shown to efficiently knockdown all five members of the miR-200 family (23) and controlled our knockdown experiment for the absence of expression of these miRs by whole-mount ISH of 2-day-old embryos (data not shown). miR-200 morphant gastrulae displayed only a small delay of epiboly progression (Fig. 7, *D* and *E*), whereas deep cell layer thinning appeared unaffected (data not shown). Furthermore, transcript levels of *zeb1a*, *zeb1b*, and *cdh1* were not affected during early segmentation (Fig. 7*F*).

In summary, our *in vivo* and *in silico* data in combination with the data by Choi *et al.* (23) indicate that the reciprocal negative feedback loop between ZEB1 and the members of the miR-200 family is conserved through evolution. However, during zebrafish gastrulation and segmentation stages, interference with the feedback loop has no major effect on morphogenesis. At this stage, miR-200s are weakly expressed (45) and may only be involved in fine-tuning of cell adhesion.

## DISCUSSION

Although many studies have shown the importance of tightly controlled cell adhesion during gastrulation, the mechanisms that accomplish this regulation are not well understood. Here, we find that Zeb1a and Zeb1b are important for control of morphogenetic cellular rearrangements during zebrafish gastrulation. We have shown that Zeb1-mediated E-cadherin repression is required for efficient modulation of cell-cell adhesion and, therefore, proper gastrulation movements. Furthermore, we identified *epcam* as a target of Zeb1b, highlighting the role of *zeb1* genes in modulating morphogenetic cell behavior through regulation of cell-cell junctions and intracellular signaling. Finally, our results show that zebrafish Zeb1 proteins control miR-200 family member expression. Together with previously published data (23) showing miR-200 regulation of *zeb1b*, this reveals that the double-negative feedback loop is conserved in evolution from zebrafish to mammals.

A tight regulation of E-cadherin expression is required for many developmental processes, whereas its deregulation is associated with pathological conditions, particularly cancer cell dissemination and subsequent metastasis. Multiple regulatory mechanisms act in concert to modulate E-cadherin function. Zebrafish *cdh1* mutants and morphants display a delay or even arrest of deep cell epiboly (18, 21, 34). During zebrafish gastrulation E-cadherin expression is controlled at the transcriptional level (39, 46), by post-translational mechanisms, including intracellular trafficking (22, 47), and by physical interactions with other proteins that affect its functionality (19).

*zeb1a* and *zeb1b* paralogous genes have similar expression patterns during zebrafish gastrulation and act partially redundant but together are indispensable for regulating morphogenetic cell behavior. Knockdown of *zeb1a* and *zeb1b* alone or together results in strongly delayed epiboly, affected emboly, and convergence movement defects. This phenotype is characteristic for zebrafish embryos with increased E-cadherin expression, like prostaglandin E synthase morphants (46, 48). *zeb1b*-overexpressing embryos display a slight delay of epiboly and affected convergence movements, phenotypes also observed for *cdh1* morphants (21, 34, 49). Our analyses of *cdh1* zygotic mRNA levels indeed showed that Zeb1b acts as a repressor of *cdh1* transcription during zebrafish gastrulation. Furthermore, cell reaggregation assays revealed that this regulation contributes to control of cell-cell adhesion. We determined whether partial knockdown of E-cadherin may ameliorate the aspects of the epiboly phenotype caused by loss of Zeb1 activity. However, combined knockdown of *cdh1* and *zeb1a/b* only marginally improved epiboly movements, suggesting that E-cadherin-mediated cell-cell adhesion is not the sole mediator of the Zeb1-dependent gastrulation defects.

Interestingly, we find Zeb1 activity to be crucial for proper zebrafish gastrulation, whereas work in *Xenopus*, chicken, and mouse have previously identified Zeb1 functions at postgastrulation stages only (50–52). Although Zeb1 is expressed in the primitive streak and ectoderm during mouse gastrulation, Zeb1 null mutants develop to birth, albeit at smaller size, and die perinatally (51, 53), suggesting that Zeb1 is not crucial for mouse gastrulation. We envision two mechanisms that may explain this discrepancy. First, Zeb1 knockdown in zebrafish prominently affects epiboly but only weakly affects emboly. Although similarities between epibolic movements during mouse and zebrafish gastrulation have been described (54), epiboly cell rearrangements occurring during zebrafish gastrulation are more prominent than in mouse, which could explain the stronger phenotype upon Zeb1 depletion in zebrafish. Second, during evolution the relative contribution of different transcription factors to regulate E-cadherin expression during gastrulation may have shifted. Snail factors, for example, are required for mesoderm delamination in *Drosophila* (55), chicken (56), and mouse (57). However in zebrafish, the down-regulation of E-cadherin by Snai1a, Snai1b, and Snai2 is dispensable for the initial steps of mesoderm internalization (39, 58, 59).

An additional level of adhesion regulation is established by the ZEB1/miR-200 feedback loop that controls cellular plasticity in cancer cells (14). A recent study revealed the importance of Zeb1/miR-200 regulation for the development of the mouse palate, which requires coordinated cellular rearrangements driven by EMT (60). So far little is known about Zeb1/miR-200 feedback loop functions during gastrulation. Our results together with previously published data (23) demonstrate that the Zeb1/miR-200 double-negative feedback loop is conserved in teleosts. However, during zebrafish gastrulation, miR-200s are expressed at relatively low levels (45) and thus appear not to be major regulators of Zeb1 expression.

We identified Epcam, a transmembrane glycoprotein mediating homophilic adhesion with expression restricted to EVL cells, as an additional Zeb1 target. *zeb1b*-overexpressing embryos display severely compromised EVL integrity, a phenotype similar to the combined MZ*epcam* mutant and *cdh1* knockdown morphant phenotype (30). Thus, E-cadherin and Epcam together appear to be prominent components of EVL cell adhesion and have the potential to be regulated by Zeb1. Interestingly, EPCAM was also shown to be involved in carcinogenesis (61). Indeed, we found a negative correlation of Zeb1b and *epcam* expression levels not only in zebrafish but also in human pancreatic (Panc-1) and breast cancer (MDA-MB231) cell lines. These findings are consistent with data showing a negative correlation between EPCAM and ZEB1 in several cancer cell lines (9, 37, 62). Beside its role as cell adhesion molecule, the intracellular domain of EPCAM (EpICD) may also act as signal transducer. In complex with FHL2 (four- and one-half LIM domains protein 2), β-catenin, and Lef-1 (lymphoid enhancer-binding factor-1), EpICD has been shown to induce the transcription of specific oncogenes, such as cyclins and c-myc, thereby promoting cancer cell proliferation (63). In *Xenopus* Epcam signaling operates through down-regulation of PKC activity, thereby regulating embryonic morphogenetic cell movements (64). In addition to direct adhesion effects, such mechanisms may explain why a relatively small decrease of zygotic *cdh1* and *epcam* levels by Zeb1b overexpression may already be sufficient to markedly affect gastrulation movements. Importantly, upon *zeb1a/b* knockdown we detected ectopic expression of *epcam* transcripts in the deep cells of the blastoderm. This result suggests that Zeb1a and Zeb1b expression in the blastoderm is necessary and sufficient to restrict the expression of *epcam* to the extra-embryonic EVL cells in WT embryos, thereby allowing gastrulation to proceed normally. In addition to *cdh1* overexpression, the ectopic expression of *epcam* in blastodermal cells may at least partially explain the strong and global gastrulation defects seen in *zeb1a/b* morphants.

**FIGURE 8.**
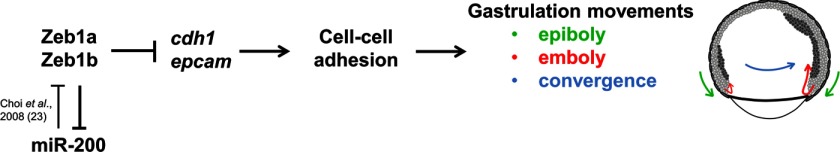
**A model of the mechanisms by which Zeb1a and Zeb1b regulate cell behavior during early zebrafish development.**

Our analysis reveals that multiple regulatory mechanisms are integrated to control cell adhesion and behavior during zebrafish gastrulation. We identified Zeb1-mediated transcriptional repression as a major mechanism to modulate newly transcribed *cdh1* during gastrulation. In the context of the vast amount of maternally derived *cdh1* mRNA and E-cadherin protein, posttranscriptional mechanisms of E-cadherin regulation, including endosomal cycling, may dominate the control of cell-cell adhesion during the early phase of gastrulation, when static blastomers become motile and epiboly is initiated (22). However, as gastrulation proceeds, transcriptional mechanisms including Zeb1-mediated repression of E-cadherin crucially contribute to modulation of the E-cadherin adhesion as well as other adhesion systems, including Epcam. It is conceivable that Zeb1 during gastrulation also regulates other targets controlling cell behavior, as shown for specific laminin (*LAMC2*) and integrin (*ITGB4*) genes in cancer cells (65). Interestingly, our data show that the miR-200 family-based feedback loop controlling Zeb1 activity is functional but does not effectively contribute to control of the Zeb1-E-cadherin regulatory system during zebrafish gastrulation and segmentation stages (Fig. 8). The strong conservation of mechanisms regulating cell adhesion during early zebrafish development and in cancer metastasis suggests that a common regulatory toolbox controls cell adhesion and EMT-like processes in development and malignant cancer progression.

## Footnotes

2 The abbreviations used are:

EMT: epithelial-mesenchymal transition
MO: morpholino
SBMO: splicing-blocking (SB) morpholino
SCMO: standard control morpholino
TBMO: translational-blocking (TB) morpholino
Epcam: epithelial cell adhesion molecule
hpf: hours post fertilization
ISH: *in situ* hybridization
miR: microRNA
MRE: miR response element
EVL: enveloping layer
qRT: quantitative real-time.
